# Association between HbA1c/HDL-C ratio and diabetic foot ulcer: a cross-sectional study based on the Han population in Northwest China

**DOI:** 10.3389/fnut.2026.1865509

**Published:** 2026-07-29

**Authors:** Jie Wang, Qiong Wang, Jianbo Sun, Yinan Jin, Hongmou Zhao

**Affiliations:** 1 Department of Foot and Ankle Surgery, Honghui Hospital, Xi’an Jiaotong University, Xi’an, Shaanxi, China; 2 Diabetic Foot MDT Center, Honghui Hospital, Xi’an Jiaotong University, Xi’an, Shaanxi, China

**Keywords:** biomarker, complication, diabetic foot ulcers, HbA1c/HDL-C ratio, nutrient metabolism

## Abstract

**Objective:**

This cross-sectional study was designed to explore the independent link between the HbA1c/high-density lipoprotein cholesterol ratio (HHR) and diabetic foot ulcer (DFU) among adult Han patients from northwest China.

**Methods:**

Clinical data from 866 patients with type 2 diabetes mellitus (T2DM) were retrospectively analyzed in Honghui Hospital of Xi’an Jiaotong University between July 2023 and March 2026. Logistic regression models were applied to estimate the association between HHR and DFU risk, with smooth curve fitting (SCF) used to characterize potential nonlinear trends. Discriminatory capacity of HHR for DFU detection was assessed using receiver operating characteristic (ROC) curve, and quantified the area under the curve (AUC). In addition, decision curve analysis (DCA) was conducted to evaluate the clinical net benefit of HHR.

**Results:**

After adjusting for all covariates, the logistic regression demonstrated that every one-unit elevation in HHR was correlated with a remarkable increase in DFU risk, corresponding to an OR of 1.327 (95% CI: 1.222–1.440). Compared to the lowest tertile, the highest tertile of HHR was linked to a substantially elevated DFU risk, with an OR of 5.366 (95% CI: 2.939–9.798). The SCF also exhibited similar trends. Moreover, compared with the triglyceride-glucose (TyG) index (AUC = 0.649, 95% CI: 0.609–0.690), the HHR yielded a significantly larger area under the ROC curve of 0.704 (95% CI: 0.663–0.745) for DFU identification. Furthermore, the results of DCA demonstrated that HHR conferred a higher net clinical benefit across a wide range of threshold probabilities for DFU identification.

**Conclusion:**

This study demonstrated a positive correlation between DFU and the combined glucose-lipid metabolic dysfunction status represented by increased HHR in northwest Chinese Han adults with T2DM, and offer supportive evidence for the promising role of HHR as an auxiliary metabolic marker for the evaluation of DFU. Accordingly, closer DFU assessment and foot examination may be essential for T2DM individuals with elevated HHR. However, additional large-sample prospective investigations are still required to validate the putative causal associations suggested by the present observational data.

## 1 Introduction

Diabetic foot ulcer (DFU) is a major contributor to disability and mortality among individuals with diabetes and remains the leading cause of non-traumatic lower-extremity amputation (1). Among patients with diabetes, the cumulative lifetime risk of DFU ranges from 19 to 34% (2), and approximately 20% of affected individuals ultimately require lower-extremity amputation (3). Notably, the 5-year mortality rate following amputation reaches an alarming 40 to 70% (3, 4). The high incidence, frequent recurrence, and substantial amputation rate of DFU impose a considerable burden on healthcare systems and reduce patients’ working capacity, thereby contributing to broader socioeconomic and public health challenges (3, 5). Evidence suggests that more than half of DFU cases and related amputations may be preventable through effective risk assessment and targeted management of high-risk individuals (6). Therefore, identifying biomarkers associated with DFU may have important clinical value for recognizing vulnerable populations and improving prevention and management strategies.

Previous studies have shown that DFU pathogenesis involves complex interactions between metabolic disturbances and inflammatory responses (7, 8). Metabolic dysfunction can initiate and sustain chronic inflammation, whereas inflammation may further aggravate metabolic imbalance. Together, these processes contribute to concurrent impairment of neural function, vascular integrity, and tissue repair, thereby promoting DFU development, persistence, and poor healing (8, 9). This interactive pathway provides a theoretical basis for developing metabolic biomarker-based tools for DFU assessment. Glycated hemoglobin (HbA1c), a product of non-enzymatic protein glycation, reflects average blood glucose levels over the preceding 2–3 months and is more stable than fasting blood glucose (FBG) (10). High-density lipoprotein cholesterol (HDL-C), an important lipid parameter, exerts several protective biological effects, including reverse cholesterol transport and anti-inflammatory activity (11). Accumulating evidence suggests that abnormal HbA1c and HDL-C levels are associated with peripheral arterial disease (PAD), peripheral neuropathy (PN), and DFU onset in patients with diabetes (12–16). However, because patients with DFU often present with concurrent glucose and lipid metabolic dysregulation, single biomarkers reflecting only one metabolic pathway may have limited utility for DFU assessment (17). In contrast, a comprehensive biomarker integrating both metabolic pathways may offer a more precise, thorough, and effective approach for the early detection of DFU.

The HbA1c/HDL-C ratio (HHR) is a novel composite biomarker that reflects integrated glucose and lipid metabolic status. This index can be calculated from routine biochemical tests and has the advantages of simplicity, low cost, and feasibility for large-scale clinical use. Recent investigations have established associations between HHR and stroke (18), major adverse cardiovascular events (19), cognitive impairment (20), non-alcoholic fatty liver disease (21), and diabetic retinopathy (22). However, no studies have explored the potential correlation between HHR and DFU. Furthermore, Northwest China exhibits relatively underdeveloped economic conditions compared to coastal, eastern, and southern regions of the country, accompanied by insufficient medical resource allocation. The region’s unique geographical and natural environment has also shaped a local dietary pattern predominantly centered on carbohydrates (23). Influenced by these factors, the number of diabetic patients in Northwest China has increased substantially from 5.13 million in 2005 to 13.79 million in 2023 (24). Previous nationwide epidemiological evidence has suggested that diabetes prevalence differs among ethnic groups in China, with the Han population showing a relatively high disease burden compared with several ethnic minority populations. This phenomenon may be related not only to genetic background, but also to population aging, urbanization, dietary transition, reduced physical activity, and socioeconomic factors (25). Given the large population size of Han adults in Northwest China and the increasing burden of diabetes in this region, further investigation of DFU-related risk factors in this population is clinically meaningful. Despite this, research focusing on DFU-related risk factors specifically targeting the Han adult population in Northwest China remains scarce.

Against this background, the present study aimed to investigate the potential association between HHR and DFU among adult Han patients with type 2 diabetes mellitus (T2DM) residing in Northwest China.

## 2 Materials and methods

### 2.1 Study population

This retrospective cross-sectional study enrolled patients diagnosed with T2DM at Honghui Hospital, Xi’an Jiaotong University, between July 2023 and March 2026. Consecutive recruitment was performed.

Inclusion criteria:

1. Diagnosed with T2DM;
2. Age ≥ 20 years;
3. Han ethnicity with at least 5 years of continuous residence in Northwest China to reduce heterogeneity caused by recent migration and to ensure relatively stable exposure to regional dietary habits and lifestyle patterns.

Exclusion criteria:

1. Thromboangiitis obliterans;
2. Pregnancy or lactation;
3. Malignancy, severe infection, or cognitive impairment;
4. Incomplete clinical or laboratory data.

The study protocol complied with the Declaration of Helsinki and was approved by the Ethics Committee of Honghui Hospital (No. 2025-KY-211-01). All data were anonymized prior to analysis.

### 2.2 Calculation of HHR

HbA1c levels were quantified in percentage (%), and HDL-C concentrations were detected in mmol/L. The formula utilized for computing the HHR is presented below:

$$\mathrm{HHR}=\frac{\mathrm{HbA}1c\;(\%)}{\mathrm{HDL}-C\;(\text{mmol}/L)}$$

In addition, we also calculated the triglyceride-glucose (TyG) index as a reference parameter for identifying DFU. Unit conversions were conducted for TG and FBG using the following conversion coefficients: 1 mmol/L TG = 88.57 mg/dL and 1 mmol/L FBG = 18 mg/dL. The TyG index was then calculated as follows (26):

$$\mathrm{TyG}=\ln\frac{\mathrm{TG}\;(\mathrm{mg}/\mathrm{dL})\times \mathrm{FPG}\;(\mathrm{mg}/\mathrm{dL})}{2}$$

### 2.3 Covariates collection

The following covariates were incorporated in the present investigation: age, sex (male/female), body mass index (BMI) (calculated as weight/height^2^), smoking status (no/yes), residential region (urban/rural), drink status (no/yes), PAD (no/yes), PN (no/yes), hypertension (no/yes), cardiovascular disease (CVD) (no/yes), estimated glomerular filtration rate (eGFR), albumin, FBG, low-density lipoprotein cholesterol (LDL-C), triglyceride (TG), uric acid, and creatinine.

### 2.4 Statistical analysis

All statistical analyses were carried out using R (version 4.5.1) along with EmpowerStats (version 2.0). *p* < 0.05 was considered statistically significant. Missing data were handled using complete-case analysis. Participants with incomplete clinical or laboratory data required for the main analyses were excluded according to the predefined exclusion criteria. Therefore, the final analytic cohort included patients with complete data for all variables incorporated into the main regression models. The normality and homogeneity of variance of the variables were evaluated using the Shapiro–Wilk test and Levene’s test, respectively. Intergroup comparisons between the DFU group and non-DFU group were performed accordingly: the Student’s t-test was utilized for variables conforming to a normal distribution, while the Wilcoxon rank-sum test was employed for variables with a non-normal distribution. In addition, the chi-square test was adopted for the comparison of categorical variables between the two cohorts. Effect sizes for baseline comparisons were assessed using standardized mean differences (SMDs). Logistic regression models were constructed to evaluate the independent association between HHR and DFU, with results expressed as odds ratios (ORs) and 95% confidence intervals (CIs). Three regression models were constructed with different covariate adjustment strategies: Model 1 was established as an unadjusted crude model. Model 2 was adjusted for age (where applicable), sex (where applicable), and body mass index (BMI). Model 3 served as the fully adjusted model, incorporating age (where applicable), sex (where applicable), BMI, residential region, smoking status, drink status, hypertension, CVD, PAD, PN, albumin, FBG, LDL-C, TG, uric acid, creatinine, and eGFR. The variance inflation factor (VIF) was used to assess multicollinearity among variables in the regression model.

Smooth curve fitting (SCF) and generalized additive models (GAMs) were used to explore nonlinear relationships. To validate the robustness of the analytical outcomes, subgroup analyses were performed by age and sex. Moreover, HHR was divided into tertiles (HHR.T), and linear trend tests were performed. We also used receiver operating characteristic (ROC) curve to evaluate the discriminative ability of HHR and TyG in DFU identification, and quantified the area under the curve (AUC). DeLong’s test was used to statistically compare the differences in AUC values between the two ROC curves. The optimum diagnostic cutoff for HHR was derived using the Youden index, and its diagnostic performance was verified by sensitivity, specificity, positive predictive value (PPV), and negative predictive value (NPV). Additionally, decision curve analysis (DCA) was applied to compare the clinical applicability and net clinical benefit of HHR and TyG for DFU risk stratification.

## 3 Results

### 3.1 Baseline characteristics

**Figure 1:**
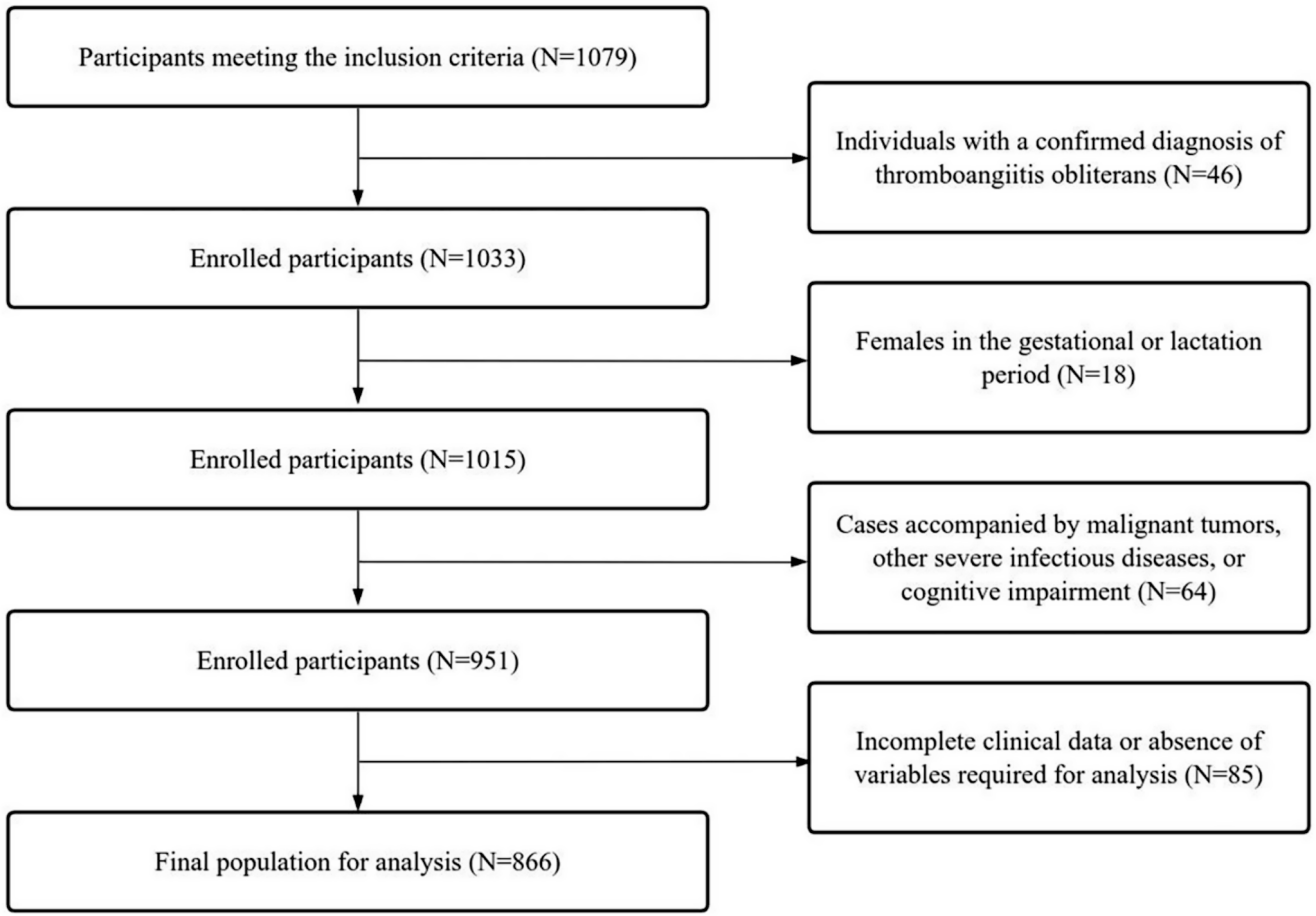
Flow diagram of inclusion criteria and exclusion criteria.

A total of 866 participants were ultimately recruited in this investigation (Figure 1). Relative to the non-DFU cohort, the DFU group showed a distinctly higher incidence of CVD (48.8% vs. 23.7%, *p < 0.001*), PAD (66.7% vs. 20.1%, *p < 0.001*) and PN (83.1% vs. 35.7%, *p < 0.001*). Additionally, creatinine (69.1 ± 28.4 vs. 80.9 ± 67.8, *p = 0.014*), eGFR (94.2 ± 11.9 vs. 102.9 ± 20.7, *p < 0.001*), albumin (39.1 ± 5.0 vs. 41.4 ± 3.6, *p < 0.001*) and HDL-C (1.0 ± 0.3 vs. 1.2 ± 0.4, *p < 0.001*) levels were markedly lower in the DFU group than in the non-DFU group. In contrast, the DFU group presented with elevated measurements of BMI (26.7 ± 4.0 vs. 24.4 ± 5.9, *p < 0.001*), HbA1c (8.7 ± 1.7 vs. 7.9 ± 1.6, *p < 0.001*), fasting plasma glucose (10.4 ± 4.9 vs. 9.4 ± 3.8, *p < 0.001*), TG (1.8 ± 0.8 vs. 1.5 ± 0.8, *p < 0.001*) and HHR (9.4 ± 3.2 vs. 7.2 ± 2.7, *p < 0.001*). Further assessments of other demographic and clinical covariates demonstrated that DFU patients were more prone to be aged 60 years and above (64.8% vs. 51.1%, *p < 0.001*), male (63.8% vs. 50.7%, *p < 0.001*) and tobacco smokers (43.7% vs. 25.1%, *p < 0.001*), as presented in Table 1.

**Table 1:** Characteristics of the study population.

Variables	Non-DFU (*N* = 653, 75.4%)	DFU (*N* = 213, 24.6%)	Effect size (SMD)	*p-*value
Age (years, %)			0.3 (0.1, 0.4)	<0.001
<60	319 (48.9%)	75 (35.2%)		
≥60	334 (51.1%)	138 (64.8%)		
Sex (%)			0.3 (0.1, 0.4)	<0.001
Male	331 (50.7%)	136 (63.8%)		
Female	322 (49.3%)	77 (36.2%)		
BMI (kg/m^2^, mean ± SD)	24.4 ± 5.9	26.7 ± 4.0	0.5 (0.3, 0.6)	<0.001
Living area (%)			0.1 (−0.1, 0.2)	0.398
Urban area	340 (52.1%)	118 (55.4%)		
Rural area	313 (47.9%)	95 (44.6%)		
Smoke (%)			0.4 (0.2, 0.6)	<0.001
No	489 (74.9%)	120 (56.3%)		
Yes	164 (25.1%)	93 (43.7%)		
Drink (%)			0.1 (−0.0, 0.3)	0.081
No	456 (69.8%)	162 (76.1%)		
Yes	197 (30.2%)	51 (23.9%)		
Hypertension (%)			0.1 (−0.1, 0.2)	0.285
No	404 (61.9%)	123 (57.7%)		
Yes	249 (38.1%)	90 (42.3%)		
CVD (%)			0.5 (0.4, 0.7)	<0.001
No	498 (76.3%)	109 (51.2%)		
Yes	155 (23.7%)	104 (48.8%)		
PAD (%)			1.1 (0.9, 1.2)	<0.001
No	522 (79.9%)	71 (33.3%)		
Yes	131 (20.1%)	142 (66.7%)		
PN (%)			1.1 (0.9, 1.3)	<0.001
No	420 (64.3%)	36 (16.9%)		
Yes	233 (35.7%)	177 (83.1%)		
HbA1c (%, mean ± SD)	7.9 ± 1.6	8.7 ± 1.7	0.5 (0.3, 0.6)	<0.001
FBG (mmol/L, mean ± SD)	9.4 ± 3.8	10.4 ± 4.9	0.2 (0.1, 0.4)	0.001
Albumin (g/L, mean ± SD)	41.4 ± 3.6	39.1 ± 5.0	0.5 (0.4, 0.7)	<0.001
Uric acid (μmol/L, mean ± SD)	313.0 ± 88.9	310.3 ± 93.6	0.0 (−0.1, 0.2)	0.712
Creatinine (μmol/L, mean ± SD)	80.9 ± 67.8	69.1 ± 28.4	0.2 (0.1, 0.4)	0.014
eGFR (mL/(min · 1.73 m^2^), mean ± SD)	102.9 ± 20.7	94.2 ± 11.9	0.5 (0.4, 0.7)	<0.001
TG (mmol/L, mean ± SD)	1.5 ± 0.8	1.8 ± 0.8	0.4 (0.2, 0.5)	<0.001
LDL-C (mmol/L, mean ± SD)	3.0 ± 0.9	3.1 ± 0.9	0.1 (−0.1, 0.2)	0.418
HDL-C (mmol/L, mean ± SD)	1.2 ± 0.4	1.0 ± 0.3	0.5 (0.4, 0.7)	<0.001
HHR (mean ± SD)	7.2 ± 2.7	9.4 ± 3.2	0.7 (0.6, 0.9)	<0.001

Effect sizes for baseline comparisons were evaluated using standardized mean differences (SMDs). Participants with incomplete clinical or laboratory data required for the main analyses were excluded according to the predefined exclusion criteria, therefore, no missing values were present for the variables included in the final analytic cohort. DFU, diabetic foot ulcer; TG, triglyceride; LDL-C, low-density lipoprotein cholesterol; HDL-C, high-density lipoprotein cholesterol; HHR, HbA1c/HDL-C ratio; FBG, fasting blood glucose; CVD, cardiovascular disease; BMI, body mass index; eGFR, estimated glomerular filtration rate; PAD, peripheral arterial disease; PN, peripheral neuropathy; SD, standard deviation; %, weighted percentage.

### 3.2 Association of HHR with DFU

#### 3.2.1 Total analyses

**Figure 2:**
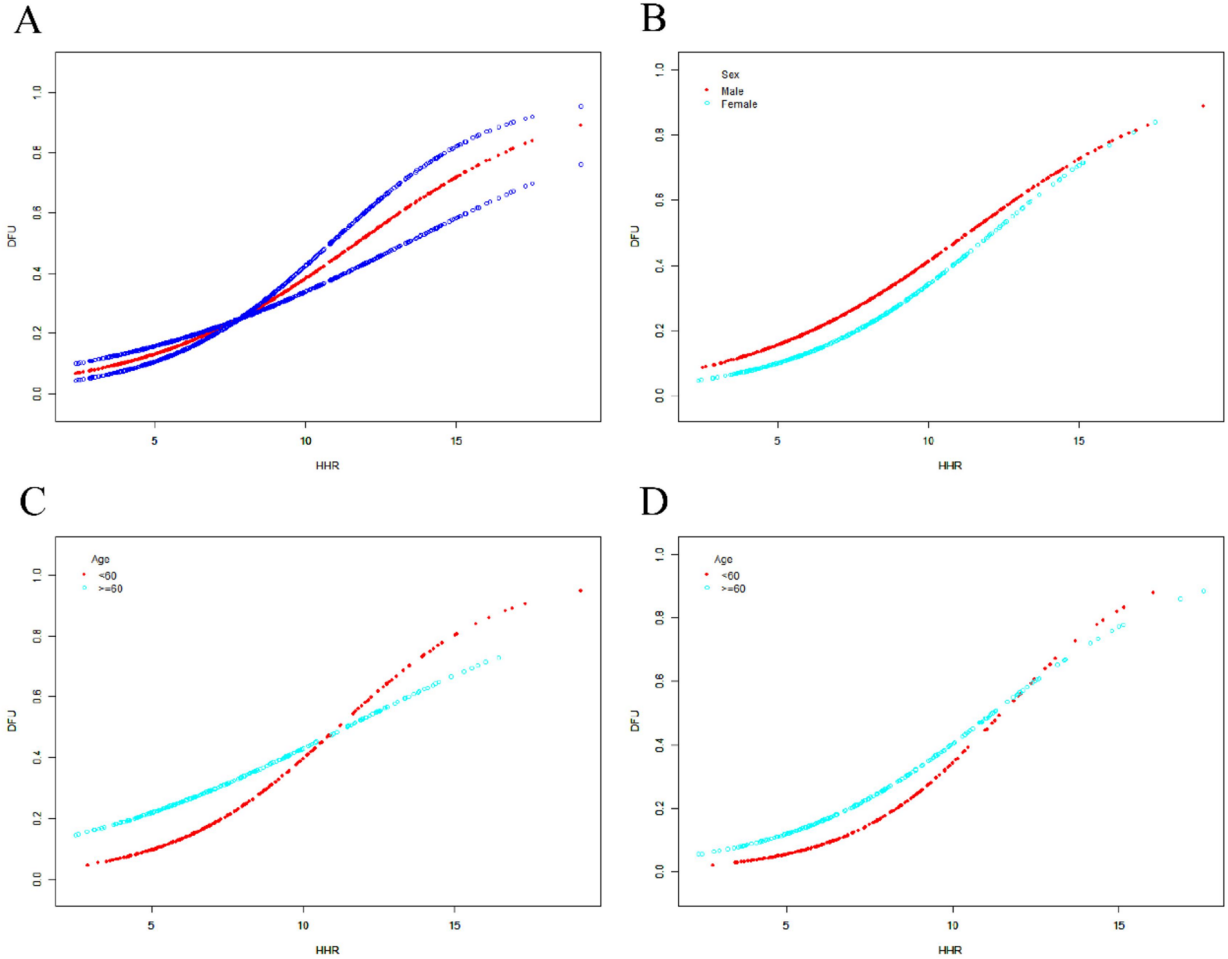
The SCF for the associations of HHR with the risk of DFU. The *x*-axis represents HHR, and the *y*-axis represents the estimated probability of DFU. **(A)** All participants were not grouped. **(B)** All participants were grouped by sex. **(C)** Male; **(D)** Female. Participants were cross-grouped by age and sex. Age (in A, B), sex (in A), BMI, residential region, smoking status, drink status, hypertension, CVD, PAD, PN, albumin, FBG, LDL-C, TG, uric acid, creatinine, and eGFR were adjusted. SCF, smooth curve fitting; DFU, diabetic foot ulcer; FBG, fasting blood glucose; TG, triglyceride; LDL-C, low-density lipoprotein cholesterol; HDL-C, high-density lipoprotein cholesterol; HHR, HbA1c/HDL-C ratio; CVD, cardiovascular disease; BMI, body mass index; eGFR: estimated glomerular filtration rate; PAD, peripheral arterial disease; PN, peripheral neuropathy.

Multivariate logistic regression analyses were performed to explore the relationship between HHR levels and the risk of DFU, and a significant positive correlation was observed in the unadjusted crude model (Model 1). Specifically, every one-unit elevation in HHR was correlated with a remarkable increase in DFU risk, corresponding to an OR of 1.261 (95% CI: 1.195–1.331). This robust positive correlation persisted after successive adjustment for age, sex, and BMI in Model 2 (OR = 1.250, 95% CI: 1.184–1.320), as well as following full adjustment for all included confounding covariates in Model 3 (OR = 1.327, 95% CI: 1.222–1.440), as presented in Table 2. In addition, results of VIF calculations showed no significant multicollinearity among variables in the regression model (Supplementary Table 1). For further analysis, HHR was categorized into three tertile groups. Relative to the lowest tertile (HHR.T1, reference group), the highest tertile of HHR (HHR.T3) was linked to a substantially elevated DFU risk in the unadjusted analysis, with an OR of 5.275 (95% CI: 3.438–8.094). After adjusting for age, sex and BMI in Model 2, the increased DFU risk in the HHR.T3 subgroup remained statistically significant compared with the HHR.T1 group (OR = 4.850, 95% CI: 3.144–7.481). Subsequent full adjustment for all potential confounding factors in Model 3 further validated this significant association; the OR for DFU risk in the HHR.T3 group was 5.366 (95% CI: 2.939–9.798) relative to the HHR.T1 group (Table 3). Importantly, linear trend tests confirmed a distinct linear correlation between ascending HHR tertiles and the probability of DFU occurrence (P for trend < 0.05, Table 3). In accordance with the above findings, the combined application of SCF and GAMs also verified a comparable positive association between HHR levels and DFU risk, as visually presented in Figure 2A.

**Table 2:** Associations of the HHR and the risk of DFU.

Exposure	Male	Female	Total
Age <60			
Model 1 OR (95% CI) *p-*value	1.297 (1.168, 1.439) < 0.001	1.387 (1.185, 1.623) < 0.001	1.325 (1.214, 1.446) < 0.001
Model 2 OR (95% CI) *p-*value	1.283 (1.154, 1.426) < 0.001	1.370 (1.171, 1.604) < 0.001	1.310 (1.199, 1.431) < 0.001
Model 3 OR (95% CI) *p-*value	1.575 (1.259, 1.970) < 0.001	2.951 (1.228, 7.094) 0.016	1.435 (1.249, 1.648) < 0.001
Age ≥60			
Model 1 OR (95% CI) *p-*value	1.213 (1.110, 1.324) < 0.001	1.232 (1.104, 1.376) < 0.001	1.220 (1.139, 1.307) < 0.001
Model 2 OR (95% CI) *p-*value	1.210 (1.106, 1.323) < 0.001	1.220 (1.092, 1.364) < 0.001	1.212 (1.130, 1.299) < 0.001
Model 3 OR (95% CI) *p-*value	1.256 (1.056, 1.493) 0.001	1.425 (1.129, 1.799) 0.003	1.266 (1.133, 1.416) < 0.001
Total			
Model 1 OR (95% CI) *p-*value	1.248 (1.167, 1.335) < 0.001	1.284 (1.173, 1.406) < 0.001	1.261 (1.195, 1.331) < 0.001
Model 2 OR (95% CI) *p-*value	1.240 (1.158, 1.328) < 0.001	1.270 (1.160, 1.391) < 0.001	1.250 (1.184, 1.320) < 0.001
Model 3 OR (95% CI) *p-*value	1.311 (1.165, 1.475) < 0.001	1.456 (1.242, 1.708) < 0.001	1.327 (1.222, 1.440) < 0.001

Model 1: no covariates were adjusted.

Model 2: age (where applicable), sex (where applicable), and BMI were adjusted.

Model 3: age (where applicable), sex (where applicable), BMI, residential region, smoking status, drink status, hypertension, CVD, PAD, PN, albumin, FBG, LDL-C, TG, uric acid, creatinine, and eGFR were adjusted.

DFU, diabetic foot ulcer; TG, triglyceride; LDL-C, low-density lipoprotein cholesterol; HDL-C, high-density lipoprotein cholesterol; HHR, HbA1c/HDL-C ratio; FBG, fasting blood glucose; CVD, cardiovascular disease; BMI, body mass index; eGFR, estimated glomerular filtration rate; PAD, peripheral arterial disease; PN, peripheral neuropathy.

**Table 3:** Association of the HHR.T and the risk of DFU.

Exposure	Male	Female	Total
Age <60			
Model 1 OR (95% CI) *p-*value			
HHR.T			
Q1	1	1	1
Q2	2.259 (0.816, 6.259) 0.117	0.473 (0.088, 2.533) 0.382	1.480 (0.657, 3.332) 0.344
Q3	7.504 (2.878, 19.564) <0.001	6.697 (2.271, 19.752) <0.001	7.073 (3.447, 14.514) <0.001
Model 2 OR (95% CI) *p-*value			
HHR.T			
Q1	1	1	1
Q2	2.080 (0.744, 5.817) 0.163	0.505 (0.093, 2.736) 0.428	1.429 (0.629, 3.246) 0.393
Q3	6.602 (2.503, 17.409) <0.001	6.126 (2.054, 18.273) 0.001	6.306 (3.049, 13.040) <0.001
Model 3 OR (95% CI) *p-*value			
HHR.T			
Q1	1	1	1
Q2	2.824 (0.537, 14.856) 0.220	2.653 (0.098, 72.095) 0.562	1.235 (0.412, 3.696) 0.706
Q3	6.420 (3.482, 18.326) 0.001	9.362 (3.361, 22.812) 0.007	7.257 (2.694, 19.546) <0.001
*P* for trend	<0.001	0.006	<0.001
Age ≥60			
Model 1 OR (95% CI) *p-*value			
HHR.T			
Q1	1	1	1
Q2	2.684 (1.233, 5.843) 0.013	1.130 (0.484, 2.637) 0.778	1.835 (1.038, 3.246) 0.037
Q3	5.826 (2.792, 12.157) <0.001	3.050 (1.396, 6.664) 0.005	4.370 (2.559, 7.462) <0.001
Model 2 OR (95% CI) *p-*value			
HHR.T			
Q1	1	1	1
Q2	2.758 (1.248, 6.096) 0.012	1.062 (0.452, 2.498) 0.889	1.782 (1.002, 3.171) 0.049
Q3	5.741 (2.710, 12.160) <0.001	2.780 (1.259, 6.139) 0.011	4.092 (2.381, 7.034) <0.001
Model 3 OR (95% CI) *p-*value			
HHR.T			
Q1	1	1	1
Q2	1.140 (0.328, 3.961) 0.837	1.068 (0.256, 4.451) 0.928	1.408 (0.623, 3.181) 0.411
Q3	4.870 (1.404, 16.898) 0.013	5.771 (1.359, 24.509) 0.018	4.893 (2.156, 11.102) <0.001
*P* for trend	0.008	0.018	<0.001
Total			
Model 1 OR (95% CI) *p-*value			
HHR.T			
Q1	1	1	1
Q2	2.487 (1.343, 4.606) 0.004	0.964 (0.463, 2.004) 0.921	1.705 (1.072, 2.711) 0.024
Q3	6.464 (3.608, 11.579) <0.001	4.069 (2.163, 7.655) <0.001	5.275 (3.438, 8.094) <0.001
Model 2 OR (95% CI) *p-*value			
HHR.T			
Q1	1	1	1
Q2	2.441 (1.306, 4.562) 0.005	0.932 (0.445, 1.949) 0.851	1.652 (1.034, 2.639) 0.036
Q3	6.078 (3.361, 10.990) <0.001	3.694 (1.949, 6.999) <0.001	4.850 (3.144, 7.481) <0.001
Model 3 OR (95% CI) *p-*value			
HHR.T			
Q1	1	1	1
Q2	1.324 (0.547, 3.206) 0.534	1.418 (0.438, 4.588) 0.560	1.384 (0.744, 2.574) 0.304
Q3	5.145 (2.165, 12.229) <0.001	8.697 (2.812, 26.899) <0.001	5.366 (2.939, 9.798) <0.001
*p* for trend	<0.001	<0.001	<0.001

Model 1: no covariates were adjusted.

Model 2: age (where applicable), sex (where applicable), and BMI were adjusted.

Model 3: age (where applicable), sex (where applicable), BMI, residential region, smoking status, drink status, hypertension, CVD, PAD, PN, albumin, FBG, LDL-C, TG, uric acid, creatinine, and eGFR were adjusted.

DFU, diabetic foot ulcer; TG, triglyceride; LDL-C, low-density lipoprotein cholesterol; HDL-C, high-density lipoprotein cholesterol; HHR, HbA1c/HDL-C ratio; HHR.T, HHR tertiles; FBG, fasting blood glucose; CVD, cardiovascular disease; BMI, body mass index; eGFR, estimated glomerular filtration rate; PAD, peripheral arterial disease; PN, peripheral neuropathy.

#### 3.2.2 Subgroup analyses

Stratified analyses stratified by age and sex further confirmed a strong positive correlation between HHR and the risk of DFU. For male participants, each 1-unit elevation in HHR was correlated with a pronounced increase in DFU risk, presenting an OR of 1.248 (95% CI: 1.167–1.335) in Model 1. This linkage remained unchanged after adjusting for age, sex and BMI in Model 2 (OR = 1.240, 95% CI: 1.158–1.328), and still existed under the full covariate adjustment condition of Model 3 (OR = 1.311, 95% CI: 1.165–1.475) (Table 2). When HHR was divided into tertiles, males in the HHR.T3 showed a significantly heightened DFU risk compared with those in the HHR.T1 in the fully adjusted Model 3, with an OR value of 5.145 (95% CI: 2.165–12.229) (Table 3). In female participants, a comparable increasing tendency of DFU risk was identified along with the rise of HHR. The OR values for each 1-unit increment of HHR were 1.284 (95% CI: 1.173–1.406) in Model 1, 1.270 (95% CI: 1.160–1.391) in Model 2 and 1.456 (95% CI: 1.242–1.708) in Model 3, respectively (Table 2). For female subjects allocated to HHR.T3, the fully adjusted Model 3 demonstrated a remarkably increased DFU risk relative to HHR.T1, with an OR of 8.697 (95% CI: 2.812–26.899) (Table 3).

Age-stratified subgroup analyses generated consistent outcomes aligned with the core findings. Among individuals younger than 60 years old, each one-unit elevation in HHR corresponded to a marked increase in DFU risk, yielding an OR of 1.325 (95% CI: 1.214–1.446) in Model 1, 1.310 (95% CI: 1.199–1.431) in Model 2, and 1.435 (95% CI: 1.249–1.648) in Model 3 (Table 2). Furthermore, the fully adjusted Model 3 indicated that subjects in the HHR.T3 group carried a considerably higher DFU risk than those in the HHR.T1 group within this younger cohort, with an OR of 7.257 (95% CI: 2.694–19.546) (Table 3). For subjects aged 60 years or older, the positive correlation between HHR and DFU risk maintained statistical significance. The ORs linked to each one-unit increment in HHR were 1.220 (95% CI: 1.139–1.307) in Model 1, 1.212 (95% CI: 1.130–1.299) in Model 2, and 1.266 (95% CI: 1.133–1.416) in Model 3 (Table 2). Within this elderly subgroup, the HHR.T3 group also presented a statistically significant increase in DFU risk relative to the HHR.T1 group in the fully adjusted Model 3, with an OR of 4.893 (95% CI: 2.156–11.102) (Table 3).

Subsequent stratified analyses integrating age and sex classifications validated that the positive association between HHR and DFU risk retained statistical significance across all combined age-sex subgroups. In detail, among males younger than 60 years of age, every 1-unit increase in HHR corresponded to a pronounced elevation in DFU risk, with an OR of 1.297 (95% CI: 1.168–1.439) in Model 1, 1.283 (95% CI: 1.154–1.426) in Model 2, and 1.575 (95% CI: 1.259–1.970) in Model 3 (Table 2); the fully adjusted multivariate Model 3 further demonstrated that the HHR.T3 category conferred a substantially increased DFU risk relative to the HHR.T1 category in this subgroup, yielding an OR of 6.420 (95% CI: 3.482–18.326) (Table 3). For males aged 60 years and older, the positive linkage remained statistically evident, as the OR values associated with each 1-unit HHR rise were 1.213 (95% CI: 1.110–1.324) in Model 1, 1.210 (95% CI: 1.106–1.323) in Model 2, and 1.256 (95% CI: 1.056–1.493) in Model 3 (Table 2); in the fully adjusted Model 3, the HHR.T3 group exhibited an OR of 4.870 (95% CI: 1.404–16.898) for DFU risk when compared with the HHR.T1 group (Table 3).

Among females below the age of 60, ascending HHR levels were likewise correlated with heightened DFU risk: the corresponding OR values per 1-unit HHR increment were 1.387 (95% CI: 1.185–1.623) in Model 1, 1.370 (95% CI: 1.171–1.604) in Model 2, and 2.951 (95% CI: 1.228–7.094) in Model 3 (Table 2), and the HHR.T3 group presented a significantly greater DFU risk than the HHR.T1 group in Model 3 (OR = 9.362, 95% CI: 3.361–22.812) (Table 3). In females aged 60 years and above, each 1-unit increment in HHR continued to correlate with an augmented DFU risk, with OR values of 1.232 (95% CI: 1.104–1.376) in Model 1, 1.220 (95% CI: 1.092–1.364) in Model 2, and 1.425 (95% CI: 1.129–1.799) in Model 3 (Table 2); the fully adjusted Model 3 revealed that the HHR.T3 group carried a 5.771-fold greater risk of DFU relative to the HHR.T1 group (95% CI: 1.359–24.509) (Table 3). Moreover, SCF and GAMs were utilized to explore the potential non-linear association between HHR and DFU risk in all aforementioned subgroups, and the corresponding outcomes are depicted in Figures 2B–D.

### 3.3 ROC curve and DCA

**Figure 3:**
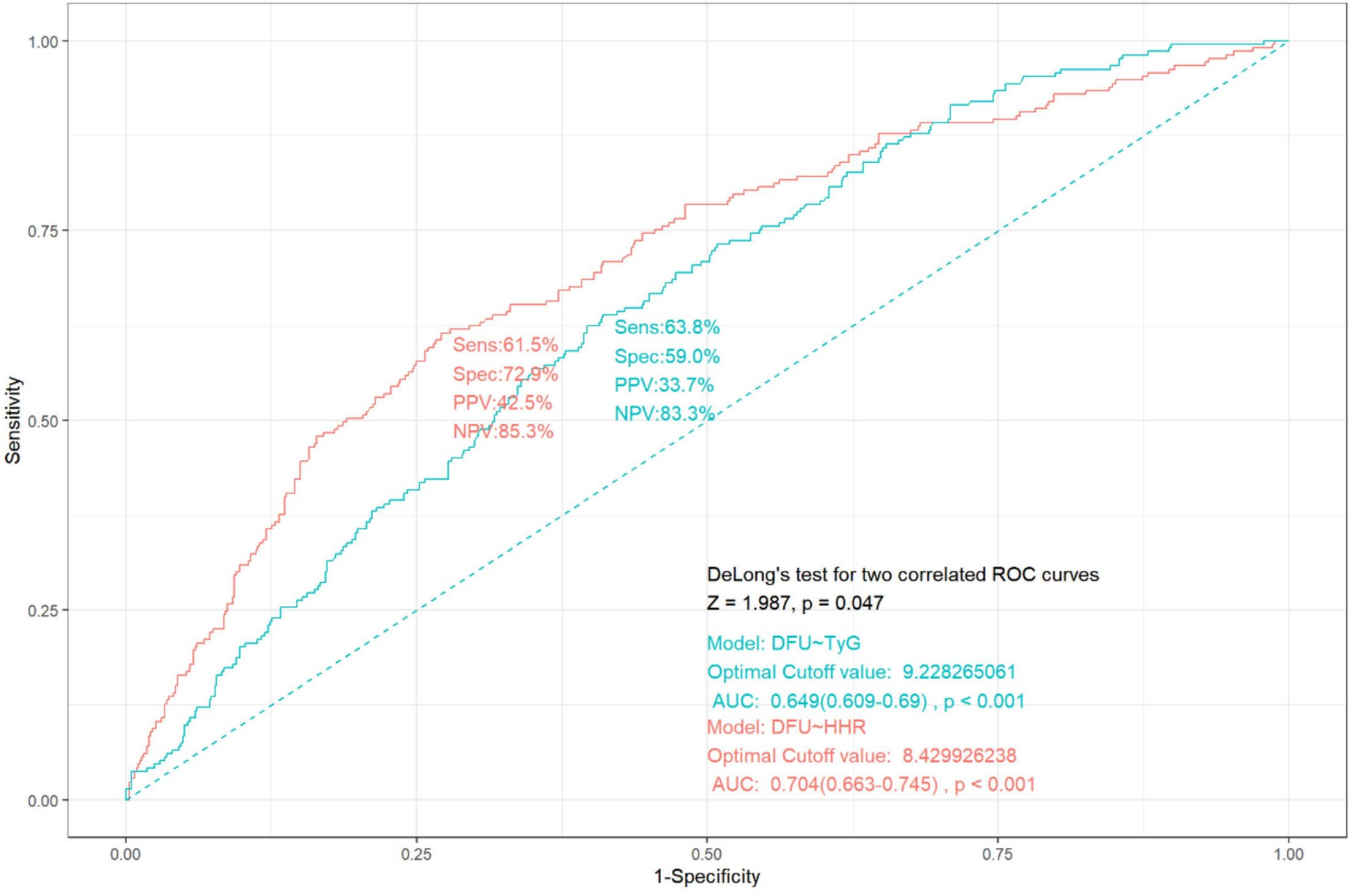
The ROC curve illustrates the performance of the HHR (red line) and TyG (green line) for DFU detection, and the AUC values were 0.704 (95% CI: 0.663–0.745) and 0.649 (95% CI: 0.609–0.690), respectively. The optimal HHR cutoff was 8.430 according to the Youden index, with a sensitivity of 61.5%, specificity of 72.9%, PPV of 42.5%, and NPV of 85.3%. The optimal TyG cutoff was 9.228 according to the Youden index, with a sensitivity of 63.8%, specificity of 59.0%, PPV of 33.7%, and NPV of 83.3%. ROC, receiver operating characteristic; DFU, diabetic foot ulcer; HDL-C, high-density lipoprotein cholesterol; HHR, HbA1c/HDL-C ratio; TyG, triglyceride-glucose; AUC, the area under the curve.

**Figure 4:**
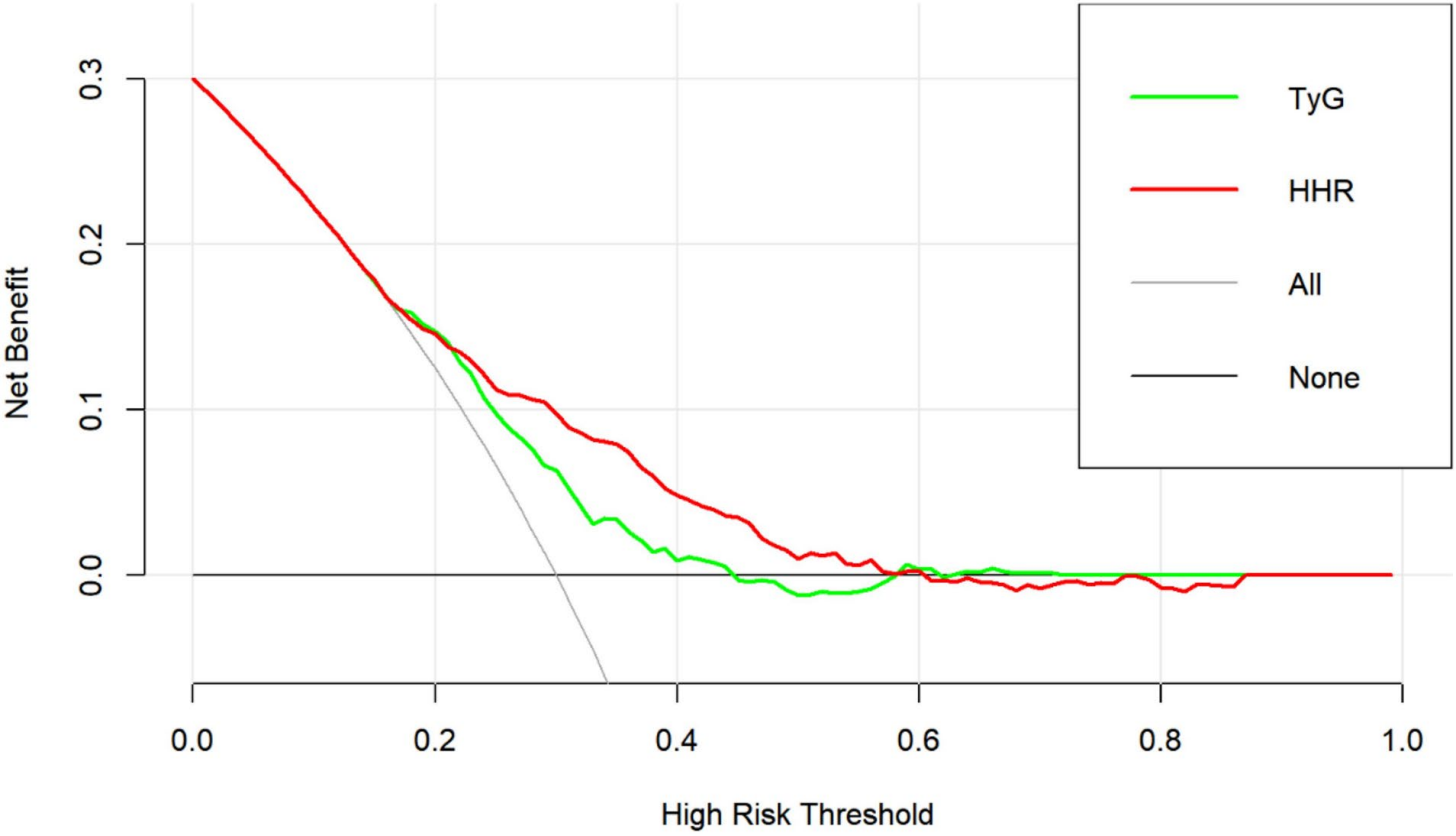
DCA of HHR and TyG for identifying DFU. The ‘treat-all’ and ‘treat-none’ strategies are shown as reference lines. The results suggest that HHR may provide greater net benefit than TyG across selected threshold probabilities in this cohort. DCA, decision curve analysis; DFU, diabetic foot ulcer; HDL-C, high-density lipoprotein cholesterol; HHR, HbA_1c_/HDL-C ratio; TyG, triglyceride-glucose.

As shown in Figure 3, compared with the TyG (AUC = 0.649, 95% CI: 0.609–0.690), the HHR yielded a significantly larger AUC of 0.704 (95% CI: 0.663–0.745) for DFU identification. DeLong’s test indicated that the difference in the AUC values between the two ROC curves was statistically significant (Figure 3, *p* = 0.047). Utilizing the maximum Youden’s index, we determined the optimal diagnostic cut-off point of HHR for DFU to be 8.430, which corresponded to a diagnostic sensitivity of 61.5% and a specificity of 72.9%. Corresponding to this critical threshold, the PPV and NPV of HHR in DFU diagnosis were computed as 42.5 and 85.3%, respectively. Furthermore, the results of DCA demonstrated that HHR conferred a higher net clinical benefit than TyG across a wide range of threshold probabilities for DFU risk stratification (Figure 4).

## 4 Discussion

In the current investigation, we detected a statistically significant positive association between HHR and the incidence of DFU among Han adults residing in Northwest China. Stratified subgroup assessments categorized by age and sex further corroborated the stability and consistency of this linkage. The findings from our work underscore the pivotal connection between the integrated glucose-lipid metabolism status and the incidence of DFU. To date, this is the first study to elucidate the potential correlation between HHR and DFU risk. Notably, this study may expand the investigative landscape of risk factors associated with DFU for the Han adult cohort in northwestern China.

In the intricate pathological course of DFU, glucose and lipid metabolic disorders exhibit an interconnected interplay coupled with a synergistic potentiation effect (8, 27). As T2DM progresses, insulin resistance promotes the activation of hormone-sensitive lipase (HSL) in adipocytes, accelerating lipolysis and increasing circulating free fatty acids (FFAs) (28–30). At the same time, reduced lipoprotein lipase (LPL) activity impairs the hydrolysis and clearance of chylomicrons and very-low-density lipoprotein (VLDL) particles (31). This process contributes to elevated TG, LDL-C, and VLDL cholesterol levels, together with reduced HDL-C levels (32). Ectopic lipid accumulation may further impair pancreatic *β*-cell function and reduce glucose uptake and utilization, thereby perpetuating a vicious cycle of glucose-lipid metabolic dysfunction (33). Chronic hyperglycemia promotes non-enzymatic glycation of endogenous proteins and lipids, leading to the formation of advanced glycation end products (AGEs) (34). Together with excess FFAs, AGEs may accumulate in the skin, vascular structures, and neural tissues of the lower extremities, thereby inducing persistent oxidative stress and chronic inflammatory responses. These pathological processes may inhibit fibroblast proliferation and collagen synthesis and contribute to peripheral neurovascular injury (34, 35), ultimately increasing susceptibility to DFU. For example, sensory neuropathy of the foot impairs pain and temperature perception, predisposing patients to unrecognized mechanical trauma and thermal injury. Motor neuropathy may cause intrinsic foot muscle atrophy and deformity, resulting in abnormal plantar pressure distribution and localized pressure overload that can promote ulcer formation. Autonomic neuropathy disrupts sweat gland function, leading to dry and fissured skin that facilitates bacterial invasion (36). In addition, PAD reduces lower-extremity perfusion and exposes foot tissues to chronic hypoxia and nutrient deprivation, which increases tissue vulnerability and impairs reparative capacity (37).

As a conventional marker of glycemic status, HbA1c has been widely implicated in diabetes-related complications, including DFU (12). In a large-scale prospective cohort study, Guo et al. (38) verified that higher HbA1c levels increase the risk of lower extremity ulcers among individuals with diabetes. Zheng et al. (39) reported that the prevalence of DFU in diabetic patients rose from 3.2 to 16.2% as the quartiles of mean HbA1c levels increased. Similarly, Dutta et al. (40) confirmed that intensive early control of HbA1c can significantly improve the healing rate of DFU. Additionally, HbA1c has been incorporated as a predictive indicator in multiple DFU-related prognostic models, which confirms its substantial value for the surveillance and identification of DFU (41, 42). Furthermore, a multitude of studies have verified that HDL-C exerts a protective role in metabolism regulation (43). For instance, a large-sample cross-sectional study conducted by Tabara et al. (44) reported a negative correlation between HDL-C levels and insulin resistance index in patients with T2DM. Specifically, HDL-C facilitates cholesterol elimination and sustains systemic lipid homeostasis by mediating reverse cholesterol transport (45). Beyond this, HDL-C counteracts hyperglycemia-induced vascular endothelial cell damage, and diminishes the likelihood of diabetic vascular complications through enhancing insulin sensitivity and optimizing pancreatic *β*-cell function (46). More importantly, the correlation between HDL-C and DFU has been thoroughly corroborated in prior research (14–16).

From a mechanistic perspective, HHR may provide integrated information on two complementary pathological processes involved in DFU. HbA1c is closely associated with AGEs, oxidative stress, endothelial dysfunction, microvascular injury, PN, impaired immune response, and delayed wound repair. These processes are central components of DFU pathophysiology (47). In contrast, HDL-C exerts several protective biological effects. The reduced HDL-C may indicate weakened lipid-related vascular and anti-inflammatory protection (44, 45). The biological significance of HHR lies in its ability to simultaneously capture increased chronic glycemic injury and decreased HDL-related protective capacity. A higher HHR may therefore represent a systemic metabolic state in which hyperglycemia-induced tissue damage occurs in parallel with impaired vascular protection and reduced anti-inflammatory reserve. This combined imbalance may promote neurovascular injury, chronic inflammation, lower-extremity ischemia, susceptibility to infection, and impaired wound healing. Accordingly, HHR may be more closely linked to the pathophysiology of DFU than HbA1c or HDL-C alone.

Moreover, we conducted comparative analyses between the HHR and the TyG index. Derived from fasting triglyceride and fasting glucose concentrations, the TyG index is a well-established surrogate marker for insulin resistance, and its relevance to a spectrum of metabolic disorders and diabetic complications has been extensively documented (48). Therefore, we selected TyG as a reference index to evaluate whether HHR could provide additional value in DFU identification. However, compared with TyG, HHR may better reflect the chronic metabolic and vascular-inflammatory background involved in DFU pathogenesis. Fasting glucose mainly represents short-term fasting metabolic status, whereas HbA1c reflects a longer-term glycemic burden (10). Chronic sustained hyperglycemia-induced glucotoxicity constitutes the core pathogenic factor of DFU. In addition, HDL-C has anti-inflammatory, antioxidative, endothelial-protective, and reverse cholesterol transport functions, all of which are biologically relevant to peripheral vascular injury and tissue repair in DFU (44, 45). Therefore, HHR may integrate long-term glycemic burden and lipid-related vascular protective capacity, making it a potentially more comprehensive marker for DFU assessment. In the present cohort, HHR showed a higher discriminatory ability for DFU than TyG, with an AUC of 0.704 (95% CI: 0.663–0.745) compared with 0.649 (95% CI: 0.609–0.690) for TyG. DeLong’s test demonstrated that this difference was statistically significant (*p* = 0.047). Furthermore, DCA indicated that HHR provided a higher net clinical benefit than TyG across a wide range of threshold probabilities. These findings support that HHR may serve as a novel biomarker for the early screening of DFU in this specific T2DM population.

Nevertheless, the present study is subject to several notable limitations. Primarily, given the retrospective design of this research, the strength of evidence for deducing a causal association between the HHR and DFU is relatively restricted. Second, although HHR demonstrated a higher AUC than the TyG index in the present cohort, its discriminatory performance was moderate. At the optimal cutoff value, the sensitivity and positive predictive value of HHR were limited, indicating that HHR alone may be insufficient for definitive DFU diagnosis or independent clinical decision-making. Therefore, HHR may be interpreted as an auxiliary metabolic indicator rather than a standalone diagnostic or screening tool. Its potential value lies in its simplicity and availability from routine laboratory tests, which may help identify patients who require closer clinical assessment, foot examination, and metabolic management. Further external validation is needed to determine whether HHR can improve DFU assessment when combined with established clinical risk factors. Third, because this was a retrospective real-world study, the number of patients in the DFU and non-DFU groups was not completely balanced, and significant baseline differences existed between the two groups. Although we adjusted for multiple demographic and clinical covariates in multivariable models and performed subgroup and sensitivity-related analyses to improve the robustness of the findings, residual confounding cannot be fully excluded. In addition, although the fully adjusted model included several demographic and clinical covariates, including age, sex, BMI, residential region, smoking status, drink status, hypertension, CVD, PAD, PN, albumin, FBG, LDL-C, TG, uric acid, creatinine, and eGFR, residual confounding cannot be completely excluded. In particular, diabetes duration and medication use, such as insulin therapy and statin treatment, were not consistently available in the retrospective dataset. These factors may influence both metabolic status and DFU development. In addition, DFU severity grading, including ulcer depth, infection status, ischemic severity, and Wagner or University of Texas classification, was not included. This study failed to conduct further stratified subgroup analyses to clarify the correlation of the HHR with distinct classifications and severity levels of DFU. Finally, this was a single-center retrospective cross-sectional study conducted in a Han Chinese population from Northwest China, which may restrict the external generalizability of the findings. Regional differences in ethnicity, lifestyle, dietary habits, socioeconomic status, diabetic foot care, medication patterns, and laboratory measurement methods may influence both HHR levels and DFU prevalence. Therefore, the association observed in the present study and the proposed HHR cutoff value should be interpreted as population-specific and exploratory. Future studies are warranted to implement independent external validation utilizing multi-center datasets from different clinical institutions and patient subgroups, with the aim of further promoting the clinical application value of this biomarker in wider medical scenarios.

## 5 Conclusion

In summary, the findings of this study indicate that elevated HHR are correlated with an increased risk of DFU in adult Han Chinese patients with T2DM residing in northwestern China. These findings support a positive correlation between DFU and the combined glucose-lipid metabolic dysfunction represented by increased HHR levels, and offer supportive evidence for the promising role of HHR as an auxiliary metabolic marker in the clinical evaluation of DFU. Accordingly, closer DFU assessment and foot examination may be essential for diabetic individuals with elevated HHR. However, additional large-sample prospective investigations are still required to validate the putative causal associations suggested by the present observational data.

## Data Availability

The raw data supporting the conclusions of this article will be made available by the authors, without undue reservation.

## References

[1] Armstrong DG Tan TW Boulton AJM Bus SA. Diabetic foot ulcers: a review. JAMA. (2023) 330:62–75. doi: 10.1001/jama.2023.10578 37395769 PMC10723802

[2] Armstrong DG Boulton AJM Bus SA. Diabetic foot ulcers and their recurrence. N Engl J Med. (2017) 376:2367–75. doi: 10.1056/NEJMra1615439 28614678

[3] McDermott K Fang M Boulton AJM Selvin E Hicks CW. Etiology, epidemiology, and disparities in the burden of diabetic foot ulcers. Diabetes Care. (2023) 46:209–21. doi: 10.2337/dci22-0043 36548709 PMC9797649

[4] Gallagher KA Mills JL Armstrong DG Conte MS Kirsner RS Minc SD . Current status and principles for the treatment and prevention of diabetic foot ulcers in the cardiovascular patient population: a scientific statement from the American Heart Association. Circulation. (2024) 149:e232–53. doi: 10.1161/cir.0000000000001192 38095068 PMC11067094

[5] Boulton AJ Vileikyte L Ragnarson-Tennvall G Apelqvist J. The global burden of diabetic foot disease. Lancet (London, England). (2005) 366:1719–24. doi: 10.1016/s0140-6736(05)67698-2 16291066

[6] Rhim B Harkless L. Prevention: can we stop problems before they arise? Semin Vasc Surg. (2012) 25:122–8. doi: 10.1053/j.semvascsurg.2012.05.002 22817863

[7] Deng H Li B Shen Q Zhang C Kuang L Chen R . Mechanisms of diabetic foot ulceration: a review. J Diabetes. (2023) 15:299–312. doi: 10.1111/1753-0407.13372 36891783 PMC10101842

[8] Jinyi L Xianguang D Yuhan D Haoyue H Zhongyu X Lianhui W . Metabolic reprogramming in diabetic foot ulcers: mechanisms, therapeutic implications and future perspectives. Metab Clin Exp. (2026) 175:156455. doi: 10.1016/j.metabol.2025.156455 41285352

[9] Lan NSR Dwivedi G Fegan PG Game F Hamilton EJ. Unravelling the cardio-renal-metabolic-foot connection in people with diabetes-related foot ulceration: a narrative review. Cardiovasc Diabetol. (2024) 23:437. doi: 10.1186/s12933-024-02527-1, 39696281 PMC11657306

[10] Su Y Xia C Zhang H Gan W Zhang GQ Yang Z . Emerging biosensor probes for glycated hemoglobin (HbA1c) detection. Mikrochim Acta. (2024) 191:300. doi: 10.1007/s00604-024-06380-7, 38709399

[11] Raja V Aguiar C Alsayed N Chibber YS ElBadawi H Ezhov M . Non-HDL-cholesterol in dyslipidemia: review of the state-of-the-art literature and outlook. Atherosclerosis. (2023) 383:117312. doi: 10.1016/j.atherosclerosis.2023.117312 37826864

[12] Casadei G Filippini M Brognara L. Glycated hemoglobin (HbA1c) as a biomarker for diabetic foot peripheral neuropathy. Diseases (Basel, Switzerland). (2021) 9:16. doi: 10.3390/diseases9010016 33671807 PMC8006047

[13] Li ZQ Zhang YP Fu GF Chen JF Zheng QP Xian XM . Evaluation of the effectiveness of diabetic foot ulcer recurrence risk prediction models: a systematic review. Iran J Public Health. (2025) 54:24–35. doi: 10.18502/ijph.v54i1.17572 39902368 PMC11787831

[14] SAY A Shirzadegan R Mousavi N Farokhi E Soleimaninejad M Jafarzadeh M. Designing a logistic regression model for a dataset to predict diabetic foot ulcer in diabetic patients: high-density lipoprotein (HDL) cholesterol was the negative predictor. J Diabetes Res. (2021) 2021:5521493. doi: 10.1155/2021/5521493 33816634 PMC7994070

[15] Deng R Tao R Luo H Gu Y Yang M Yin C. Association of high-density lipoprotein-related inflammatory indicators with diabetic foot ulcer in patients with diabetes: a population-based study. Diabetol Metab Syndr. (2025) 17:369. doi: 10.1186/s13098-025-01962-8, 40988077 PMC12455812

[16] Chen L Ma W Chen D Wang C Gao Y Ran X. Association of high-density lipoprotein cholesterol and wound healing in patients with diabetic foot ulcers. Chin Med J. (2022) 135:110–2. doi: 10.1097/cm9.0000000000001544 33950872 PMC8850818

[17] Wang Y Shao T Wang J Huang X Deng X Cao Y . An update on potential biomarkers for diagnosing diabetic foot ulcer at early stage. Biomed Pharmacother. (2021) 133:110991. doi: 10.1016/j.biopha.2020.110991 33227713

[18] Huang C You H Zhang Y Fan L Feng X Shao N. Association between the hemoglobin A1c/high-density lipoprotein cholesterol ratio and stroke incidence: a prospective nationwide cohort study in China. Lipids Health Dis. (2025) 24:25. doi: 10.1186/s12944-025-02438-4, 39863906 PMC11762894

[19] Huang J Zhang J Xie Z Li L Chen M Li Y . Integrated metabolic-inflammatory risk assessment: HbA1c/HDL-c and hsCRP-to-albumin ratios synergistically predict major adverse cardiovascular events in post-PCI STEMI patients. Lipids Health Dis. (2025) 24:280. doi: 10.1186/s12944-025-02707-2, 40940664 PMC12433004

[20] Chen J Zhu L Liu J Chen J Liang C Liu C . Longitudinal investigation of the HbA1c/HDL-c ratio as a potential biomarker of cognitive decline among Chinese older adults: evidence from CHARLS. BMC Public Health. (2025) 25:4138. doi: 10.1186/s12889-025-25367-4, 41291653 PMC12648874

[21] Wu J Yu W Huang L Hou S Huang Y Huang Z . The HbA1c/HDL-C ratio as a screening indicator of NAFLD in U.S. adults: a cross-sectional NHANES analysis (2017-2020). BMC Gastroenterol. (2025) 25. doi: 10.1186/s12876-025-03974-0, 40369422 PMC12076897

[22] Wang S Pan X Zhang M Chen S. Correlation between glycolipid metabolism levels and diabetic retinopathy in patients with type 2 diabetes mellitus. Diabetes Metab Syndr Obes. (2024) 17:1–9. doi: 10.2147/dmso.S437586 38192497 PMC10771718

[23] Xiang Y Ren B Chen Y Jiang M Wang N Niu R . Predictors of glycemic control among patients with type 2 diabetes in Western China: a multi-center cross sectional study. Biol Pharm Bull. (2021) 44:620–6. doi: 10.1248/bpb.b20-00898 33952818

[24] Zhou YC Liu JM Zhao ZP Zhou MG Ng M. The national and provincial prevalence and non-fatal burdens of diabetes in China from 2005 to 2023 with projections of prevalence to 2050. Mil Med Res. (2025) 12:28. doi: 10.1186/s40779-025-00615-1 40457495 PMC12128495

[25] Li Y Teng D Shi X Qin G Qin Y Quan H . Prevalence of diabetes recorded in mainland China using 2018 diagnostic criteria from the American Diabetes Association: national cross sectional study. BMJ. (2020) 369:m997. doi: 10.1136/bmj.m997 32345662 PMC7186854

[26] Huo RR Liao Q Zhai L You XM Zuo YL. Interacting and joint effects of triglyceride-glucose index (TyG) and body mass index on stroke risk and the mediating role of TyG in middle-aged and older Chinese adults: a nationwide prospective cohort study. Cardiovasc Diabetol. (2024) 23:30. doi: 10.1186/s12933-024-02122-4, 38218819 PMC10790273

[27] Li X Wen S Dong M Yuan Y Gong M Wang C . The metabolic characteristics of patients at the risk for diabetic foot ulcer: a comparative study of diabetic patients with and without diabetic foot. Diabetes Metab Syndr Obes Targets Ther. (2023) 16:3197–211. doi: 10.2147/dmso.S430426 PMC10590077 37867628

[28] Dufau J Recazens E Bottin L Bergoglio C Mairal A Chaoui K . Nuclear hormone-sensitive lipase regulates adipose tissue mass and adipocyte metabolism. Cell Metab. (2025) 37:2250–2263.e9. doi: 10.1016/j.cmet.2025.09.014 41135514

[29] Osório J. Diabetes: absence of hormone-sensitive lipase associated with risk of T2DM. Nat Rev Endocrinol. (2014) 10:445. doi: 10.1038/nrendo.2014.92 24913519

[30] Yu HC Jeon YG Na AY Han CY Lee MR Yang JD . P21-activated kinase 4 counteracts PKA-dependent lipolysis by phosphorylating FABP4 and HSL. Nat Metab. (2024) 6:94–112 eng. doi: 10.1038/s42255-023-00957-x 38216738

[31] Filali-Mouncef Y Hunter C Roccio F Zagkou S Dupont N Primard C . The ménage à trois of autophagy, lipid droplets and liver disease. Autophagy. (2022) 18:50–72 eng. doi: 10.1080/15548627.2021.1895658, 33794741 PMC8865253

[32] Kane JP Pullinger CR Goldfine ID Malloy MJ. Dyslipidemia and diabetes mellitus: role of lipoprotein species and interrelated pathways of lipid metabolism in diabetes mellitus. Curr Opin Pharmacol. (2021) 61:21–7 eng. doi: 10.1016/j.coph.2021.08.013 34562838

[33] Tang Y Majewska M Leß B Mehmeti I Wollnitzke P Semleit N . The fate of intracellular S1P regulates lipid droplet turnover and lipotoxicity in pancreatic beta-cells. J Lipid Res. (2024) 65:100587. doi: 10.1016/j.jlr.2024.100587, 38950680 PMC11345310

[34] Khalid M Petroianu G Adem A. Advanced glycation end products and diabetes mellitus: mechanisms and perspectives. Biomolecules. (2022) 12. doi: 10.3390/biom12040542, 35454131 PMC9030615

[35] Khoi CS Lin TY Chiang CK. Targeting insulin resistance, reactive oxygen species, inflammation, programmed cell death, ER stress, and mitochondrial dysfunction for the therapeutic prevention of free fatty acid-induced vascular endothelial lipotoxicity. Antioxidants (Basel). (2024) 13:1486. doi: 10.3390/antiox13121486, 39765815 PMC11673094

[36] Sloan G. Selvarajah D. Tesfaye S. Pathogenesis, diagnosis and clinical management of diabetic sensorimotor peripheral neuropathy. Nat Rev Endocrinol (2021);17:400–420. eng. doi: 10.1038/s41574-021-00496-z. 34050323

[37] Meloni M Vas PRJ. Peripheral arterial disease in diabetic foot: one disease with multiple patterns. J Clin Med. (2025) 14. doi: 10.3390/jcm14061987, 40142794 PMC11942964

[38] Guo G Guan Y Chen Y Ye Y Gan Z Cao X . HbA1c and the risk of lower limb ulcers among diabetic patients: an observational and genetics study. J Diabetes Res. (2025) 2025:4744194. doi: 10.1155/jdr/4744194 40190410 PMC11972128

[39] Zheng Z Cao B Ke J Zhao D. The effect of long-term glycemic burden on the incidence of diabetic foot ulcers: a retrospective study. J Diabetes Complicat. (2025) 39:108901. doi: 10.1016/j.jdiacomp.2024.108901, 39693944

[40] Dutta A Bhansali A Rastogi A. Early and intensive glycemic control for diabetic foot ulcer healing: a prospective observational nested cohort study. Int J Low Extrem Wounds. (2023) 22:578–87. doi: 10.1177/15347346211033458 34279130

[41] Wang M Chen D Fu H Xu H Lin S Ge T . Development and validation of a risk prediction model for the recurrence of foot ulcer in type 2 diabetes in China: a longitudinal cohort study based on a systematic review and meta-analysis. Diabetes Metab Res Rev. (2023) 39. doi: 10.1002/dmrr.3616, 36657181

[42] Zhou Z Jia Y Yan H Xu J Wang S Wen J. Risk prediction models for patients with recurrent diabetic foot ulcers: a systematic review. Public Health. (2025) 244:105744. doi: 10.1016/j.puhe.2025.105744 40347679

[43] Kardassis D Thymiakou E Chroni A. Genetics and regulation of HDL metabolism. Biochim Biophys Acta Mol Cell Biol Lipids. (2022) 1867:159060. doi: 10.1016/j.bbalip.2021.159060 34624513

[44] Tabara Y Arai H Hirao Y Takahashi Y Setoh K Kawaguchi T . Different inverse association of large high-density lipoprotein subclasses with exacerbation of insulin resistance and incidence of type 2 diabetes: the Nagahama study. Diabetes Res Clin Pract. (2017) 127:123–31. doi: 10.1016/j.diabres.2017.03.018 28365559

[45] He W Wang M Zhang X Wang Y Zhao D Li W . Estrogen induces LCAT to maintain cholesterol homeostasis and suppress hepatocellular carcinoma development. Cancer Res. (2024) 84:2417–31. doi: 10.1158/0008-5472.Can-23-3966 38718297

[46] Rotllan N Julve J Escolà-Gil JC. Type 2 diabetes and HDL dysfunction: a key contributor to glycemic control. Curr Med Chem. (2024) 31:280–5. doi: 10.2174/0929867330666230201124125 36722477

[47] Yu MG Gordin D Fu J Park K Li Q King GL. Protective factors and the pathogenesis of complications in diabetes. Endocr Rev. (2024) 45:227–52. doi: 10.1210/endrev/bnad030 37638875 PMC10911956

[48] Sun Y Ji H Sun W An X Lian F. Triglyceride glucose (TyG) index: a promising biomarker for diagnosis and treatment of different diseases. Eur J Intern Med. (2025) 131:3–14. doi: 10.1016/j.ejim.2024.08.026 39510865

