# Supplementary material for: Association between HbA1c/HDL-C ratio and diabetic foot ulcer: a cross-sectional study based on the Han population in Northwest China
Source: Front Nutr. 2026 Jul 29;13:1865509. doi: 10.3389/fnut.2026.1865509 (PMC13462043; doi:10.3389/fnut.2026.1865509)
Supplement: Supplementary file 1 [file Table_1.docx]

Supplementary Table 1

|  | **GVIF** | **Df** | **GVIF^(1/(2*Df))** |
| --- | --- | --- | --- |
| **Age** | 1.047512 | 1 | 1.023480 |
| **Sex** | 1.834337 | 1 | 1.354377 |
| **BMI** | 1.070671 | 1 | 1.034732 |
| **Living area** | 1.425713 | 1 | 1.194032 |
| **Smoke** | 1.271636 | 1 | 1.127668 |
| **Drink** | 1.076787 | 1 | 1.037683 |
| **Hypertension** | 1.076620 | 1 | 1.037603 |
| **CVD** | 1.076749 | 1 | 1.037665 |
| **PAD** | 1.187986 | 1 | 1.089948 |
| **PN** | 1.098834 | 1 | 1.048253 |
| **FBG** | 1.072632 | 1 | 1.035679 |
| **TG** | 1.100504 | 1 | 1.049049 |
| **Albumin** | 1.152139 | 1 | 1.073377 |
| **Uric acid** | 1.036988 | 1 | 1.018326 |
| **Creatinine** | 1.073212 | 1 | 1.035959 |
| **eGFR** | 1.096680 | 1 | 1.047225 |
| **LDL-C** | 1.038928 | 1 | 1.019278 |
| **HHR** | 1.185273 | 1 | 1.088702 |

TG, triglyceride; LDL-C, low-density lipoprotein cholesterol; HDL-C, high-density lipoprotein cholesterol; HHR, HbA1c/HDL-C ratio; FBG, fasting blood glucose; CVD, cardiovascular disease; BMI, body mass index; eGFR, estimated glomerular filtration rate; PAD, peripheral arterial disease; PN, peripheral neuropathy; GVIF, generalized variance inflation factor; Df, degree of freedom
